# Time-series prediction of settlement deformation in shallow buried tunnels based on EMD-SSA-GRNN model

**DOI:** 10.1038/s41598-024-51165-w

**Published:** 2024-01-03

**Authors:** Tao Li, Jiajun Shu, Duliang Chang

**Affiliations:** School of Mechanics and Civil Engineering, China University of Mining and Technology-Beijing, Beijing, 100010 China

**Keywords:** Civil engineering, Natural hazards

## Abstract

Tunnel settlement deformation monitoring is a complex task and can result in nonlinear dynamic changes. To overcome the disturbances caused by historical data and the difficulty in selecting input parameters during deformation prediction, a decomposition, reconstruction and optimization method for tunnel settlement deformation prediction is proposed. First, empirical mode decomposition (EMD) is used to decompose the in-situ monitoring data and reduce the interactions among information at different scales in sequences. Then, the monitoring data are decomposed into intrinsic mode functions (IMFs). Secondly, the smoothing factor of the generalized regression neural network (GRNN) is optimized by using the sparse search algorithm (SSA). An EMD-SSA-GRNN deformation prediction model is developed using the optimized GRNN algorithm and is used to predict the changes in the decomposed IMFs. Finally, using the measured deformation data from a shallowly buried tunnel along the Kaizhou-Yunyang Highway in Chongqing, China, the reliability and accuracy of different models are analysed. The results show that tunnel settlement deformation exhibited a trend and a slow change in the early stage, a rapid change in the middle stage and a slow change in the late stage, and the rate of change was significantly influenced by the excavation time and the upper and lower geological layers. The prediction accuracy of the EMD-SSA-GRNN model after EMD improved from 19.2 to 59.4% relative to that of the SSA-GRNN and single GRNN models. Moreover, we find that the three error evaluation indicators of the EMD-SSA-GRNN model are lower than those of the other models and that the results of the proposed model and are more strongly correlated with measured data.

## Introduction

The geological conditions in China are complex and variable, with mountainous and hilly terrain. The interiors of road tunnels located in mountainous areas contain many rock bodies with heavily developed joints and fissures. This in turn leads to issues such as instability of the surrounding rock, which can result in tunnel collapses. The control of settlement deformation in tunnel engineering is a key factor in ensuring construction safety, and the development and trend of settlement deformation during the long-term construction of a tunnel can be mitigated with appropriate control. Therefore, carrying out research on the prediction of tunnel settlement and deformation time series is highly practical for engineering safety.

To overcome the shortcomings of traditional methods in the temporal prediction of tunnel deformation, we can apply intelligent algorithms to improve the traditional prediction models^1–6^. Intelligent algorithms are capable of self-optimizing measured tunnel settlement deformation data and fitting complex nonlinear relationships. These algorithms include back propagation (BP), support vector machine (SVM), particle swarm optimization (PSO), and artificial neural network (ANN) algorithms^7–12^. Moreover, many algorithms have certain defects, and the existence of such defects decreases the calculation accuracy. For example, the BP algorithm is significantly affected by the initial parameters and may yield a local optimal solution. The parameters of the SVM algorithm are difficult to select, and the network structure is complex, so its operation efficiency is low^13,14^. Based on the development of the neural network algorithm, scholars constructed a generalized regression neural network (GRNN) algorithm with powerful analytical capabilities. The GRNN has significant advantages in parameter optimization, kernel function selection and sample processing. Moreover, the value of the smoothing factor for a single parameter is empirical, so the GRNN algorithm is widely used in foundation pit displacement prediction, surface settlement prediction and other related tasks^15–20^. At the same time, scholars have targeted the optimization of intelligent algorithms applied for deformation prediction. The core of algorithmic optimization is the use of neural network elements to construct a deformation prediction model for AI-based assessment^21,22^. For instance, Zhang et al.^17^ and Zhang et al.^23^ optimized the smoothing factor in the GRNN algorithm using the particle swarm optimization (PSO) algorithm and fruit fly optimization algorithm (FOA), respectively. The impact of the empirical selection of parameters such as the smoothing factor on the model accuracy and generalization ability was reduced with this approach, which in turn improved the prediction accuracy of the model. Shen et al.^24^ and Elbaz et al.^25,26^ predicted the energy consumption of the cutter drive process in shield tunnelling and the trajectory of shield movement during tunnelling with CNN and LSTM methods, respectively. Moreover, engineering researchers have shown that the neural network method can be effectively used to predict the displacement changes in a shield during the process of tunnel construction.

The actual tunnel construction process is long. The surrounding rock deformation fluctuates widely and is obviously affected by various factors. When an intelligent optimization algorithm is directly trained on initial deformation data and used to make predictions, certain problems, such as slow data convergence, poor fitting of wave segments, and prediction distortion, can easily occur. If the initial data are smoothed or denoised in advance, it is easy to lose some information associated with the data, which reduces the practicality of the prediction results. Therefore, we can first decompose complex time series into IMFs to reduce the complexity of the time series^27,28^. Second, we can use a neural network algorithm to optimize the IMFs and improve the accuracy of predictions. Finally, we can superimpose the prediction components to obtain the prediction result, which reduces the prediction error. At present, commonly used time–frequency analysis methods include short-time Fourier transform (STFT), wavelet transform (WT) and empirical mode decomposition (EMD). Compared to the STFT and WT methods, EMD can be utilized to decompose complex time series into relatively simple intrinsic mode components and thus reduce the data component mixing phenomenon; this approach is commonly applied for nonsmooth signals.

Based on the brief introduction above, we know that the EMD, SSA and GRNN methods have different purposes in data analysis. Specifically, the EMD method is used to reduce the influence of different types of scale information in a data series, and the GRNN is used to perform deep learning of the data components. However, if we directly use the GRNN model to analyse settlement deformation data, oscillations and dispersion may occur in the analysis results. There is an urgent need for an optimization method that can correlate different influencing factors. This method can improve the prediction accuracy and reliability of GRNNs and overcome the defects introduced by the direct optimization process. In this paper, we focus on the real-time deformation monitoring of a shallow buried tunnel along the Kaizhou-Yunyang Highway in Chongqing, China, as an example. First, we utilize EMD to disaggregate the initial data obtained from monitoring, thus reducing the interaction effects of information at different scales over time. Second, we perform the comprehensive learning of different components via the GRNN and optimize the smoothing coefficients with the SSA. Finally, we establish a decomposition-prediction-reconstruction tunnel deformation model based on EMD-SSA-GRNN. We compare our model with other prediction models to verify its reliability and practicality.

The conclusions of this study can be used to prevent sudden changes caused by the short-term rapid construction of shallow buried tunnels and for the prediction of future settlement and deformation. Moreover, we introduce the SSA to optimize the key parameter smoothing factor of the GRNN, which is difficult to subjectively set, to reduce the adverse effects of artificially determined parameters on the prediction accuracy and generalizability of the model. The aim of this study is to provide a reference basis for deformation prediction during the long-term construction of shallow buried tunnels.

## Principle of the EMD-SSA-GRNN model

The deformation of shallow buried tunnels is mainly affected by two main factors: geological conditions and external effects. The geological conditions include the topography, geomorphology, lithology, etc., and the external effects include rainfall, construction conditions, measurement conditions, etc.^29^. Tunnel deformation is a time-dependent displacement term under geological conditions and a random displacement term under external effects. The displacement trend is a smooth series that reflects the main pattern of deformation. Random displacement is a smooth noise sequence, and this sequence reduces the realism and accuracy of deformation prediction. To accurately represent the variation in each displacement component, we analyse tunnel settlement deformation data using a time series principle, as shown in Eq. (1)^30^:

1 $$s(t) = \alpha (t) + \beta (t),$$

where *s*(*t*) is the total displacement of tunnel settlement deformation, *α*(*t*) is the trend displacement, and *β*(*t*) is the random displacement.

Since most signals can be split into intrinsic mode components, we use EMD to decompose a nonsmooth and nonlinear time series into multiple smooth and linear IMFs^31^. The IMFs have essentially the same number of zeros and local extrema over the entire time series range, so the sum of the local maxima of the upper envelope and the local minima of the lower envelope of the IMFs is zero. To decompose the input signal via EMD, we need to create the time series function *X*(*t*) over a given period. After obtaining the function *X*(*t*), we use a cubic spline curve to connect the local maxima and local minima of the time series function *X*(*t*) to form the upper envelope *O*_+_(*t*) and the lower envelope *O*_-_(*t*), respectively. Ultimately, it is possible to include all the input data points between the upper and lower envelopes. Accordingly, we obtain the mean envelope *m*_1_(*t*) of the function *X*(*t*).

2 $$m_{1} (t) = \frac{{o_{ + } (t) + o_{ - } (t)}}{2}.$$

The time series function *X*_1_(*t*) is subtracted from the mean envelope *m*_1_(*t*) to obtain the first intrinsic mode component *h*_1_(*t*).

3 $$h_{1} (t) = X_{1} (t) - m_{1} (t).$$

If *h*_1_(*t*) does not satisfy the IMFs, we repeat the above steps to continue the decomposition to obtain *h*_2_(*t*). This process is continued until we obtain *h*_*k*_(*t*) satisfying the conditions defined by the IMFs. At this point, the first *IMF*_1_(*t*) is obtained.

4 $$h_{1} (t) = IMF_{1} (t).$$

A new time series function *X*_2_(*t*) is obtained by subtracting the intrinsic mode component *h*_1_(*t*) from the time series function *X*_1(_*t*).

5 $$X_{2} (t) = X_{1} (t) - IMF_{1} (t).$$

The decomposition behaviour is repeated until *X*_*n*_(*t*) becomes a monotonic function, at which point there are no longer IMFs of *X*_*n*_(*t*).

6 $$X(t) = \sum\limits_{i = 1}^{n - 1} {IMF_{n - 1} (t)} + X_{n} (t).$$

For the target signal of modal superposition, the first IMF obtained from its decomposition mainly represents the most complex state in the superposition. Moreover, the energy generated by the superposition is much greater than the modal energy contained in the IMFs of the later sequences. First, the reconstructed target signal is obtained by superimposing all the IMFs other than the first IMFs. Second, these samples from the first IMF are circularly shifted to obtain different samples. These samples are subsequently superimposed on the remaining IMFs to obtain a noisy target signal with a constant signal-to-noise ratio. In the newly obtained noise-containing signal, the target signal remains almost unchanged, while the power of the Gaussian white noise is largely attenuated. Repeating the above steps several times can gradually weaken the power of the noise signal. Finally, the noise is further suppressed using a method similar to wavelet soft threshold denoising.

Immediately afterwards, we need to add the SSA to the GRRN. The SSA is inspired mainly by the foraging behaviour and antipredation behaviour of sparrows when searching for the optimal solution. The essence of SSA optimization is to gradually find the position corresponding to the highest food energy, i.e., to find the optimal solution of the objective, by updating and changing the positions of the discoverers, followers, and alerters in the sparrow population in each generation^32^. It is assumed that the sparrow population is *X* = [*x*_1_,* x*_2_,···, *x*_*n*_]^*T*^ and that *n* is the number of sparrows. The individuals in the d-dimensional SSA are initialized as *X*_*i*_ = [*x*_*i,*1_,* x*_*i,*2_,···, *x*_*i,d*_]. The positions of the discoverers, followers and alerters are calculated for each iteration of the update process, as shown in Eqs. (7), (8) and (9), respectively^33^.

7 $$X_{i,j}^{t + 1} = \left\{ {\begin{array}{*{20}c} {X_{ij} \exp \left( { - \frac{i}{{a \cdot iter_{\max } }}} \right)} & {R_{2} < ST} \\ {X_{ij} + QL} & {R_{2} \ge ST} \\ \end{array} } \right.,$$

8 $$X_{i,j}^{t + 1} = \left\{ {\begin{array}{*{20}c} {Q\,\,\exp \left( {\frac{{X_{{{\text{worst}}}} - X_{i,j}^{t} }}{{i^{2} }}} \right)} & {i > n/2} \\ {X_{P}^{t + 1} + \left| {X_{ij} - X_{P}^{t + 1} } \right|A^{ + } L} & {otherwise} \\ \end{array} } \right.,$$

9 $$X_{i,j}^{t + 1} = \left\{ {\begin{array}{*{20}c} {X_{{{\text{best}}}}^{T} + \beta \left| {X_{ij}^{t} - X_{{{\text{best}}}}^{t} } \right|} & {if \, f_{i} > f_{g} } \\ {X_{i,j}^{t + 1} + K\frac{{\left| {X_{i,j}^{t} - X_{{{\text{worst}}}}^{t} } \right|}}{{\left( {f_{i} - f_{w} } \right) + \varepsilon }}} & { \, if \, f_{i} = f_{g} } \\ \end{array} } \right.,$$

where *iter*_max_ is the maximum number of iterations. *a* is a random number between [0, 1]. *Q* is a random number obeying a normal distribution. *L* is a 1 × *d* dimensional matrix, the range of *R*_2_ is [0, 1] as the warning value, and the range of *ST* is [0.5, 1] as the safety value. *X*_worst_ is the worst position of a sparrow at the tth iteration in *d* dimensions, and *X*_*p*_ is the optimal position of the sparrow at iteration *t* + 1 in *d* dimensions, *A* denotes a matrix of order 1 × *d*, and *A*^+^ = *A*^*T*^*(AA*^*T*^*)*^*-1*^. *X*_best_ is the current globally optimal position. *β* and *K* are the step control parameters. *β* is a normally distributed random number with a mean of 0 and a variance of 1. *K* is [0, 1] and is the moving direction of the sparrow. *ε* is a very small number to prevent a denominator of 0 from occurring. *f*_*i*_, *f*_*g*_, and *f*_*w*_ are the current individual sparrow value, optimal adaptation value and worst adaptation value, respectively.

From Eq. (7), we can find that if *R*_2_ < ST, the finder will continue its search for the optimal location and fly to another safe place to forage. This step improves the global and local search capabilities of the algorithm. From Eq. (8), if *i* > *n*/2, the current position is very poor, and other positions need to be analysed. Then, the discoverer continues to explore. During this step, the follower has the possibility of becoming a new discoverer. This step can improve the iteration speed of the algorithm. Equation (9) shows that when *f*_*i*_ ≠ *f*_*g*_, the sparrow is at the edge of the population, and the sparrow moves to the optimal position. When *f*_*i*_ = *f*_*g*_, the sparrow is at the global optimal position, and the current position is not ideal; thus, movement to another position is needed. This step can prevent the algorithm from converging to a local optimal solution.

**Figure 1:**
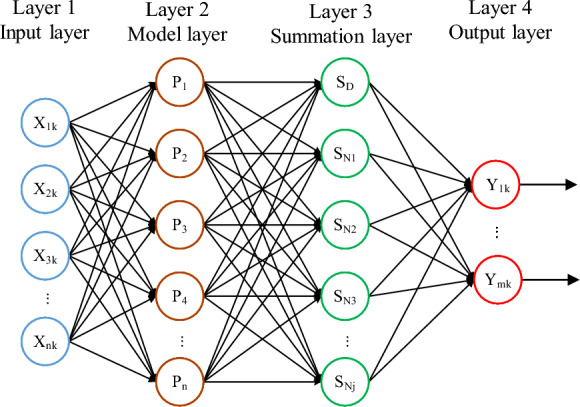
Topology of the GRNN.

The theoretical basis of the GRNN is nonlinear regression analysis. With this basis, the GRNN does not have to perform iterative calculations and can directly use the independent variables to find the regression value of the dependent variable. As shown in Fig. 1, the top-step structure of the GRNN consists of an input layer, a model layer, a summation layer and an output layer, and the operation process is shown in Eq. (10).

10 $$\left\{ \begin{gathered} P_{i} = \exp \left[ { - \frac{{(X - X_{i} )^{T} (X - X_{i} )}}{{2\sigma^{2} }}} \right] \hfill \\ S_{D} = \sum\limits_{i = 1}^{i = n} {P_{i} } ,S_{Nj} = \sum\limits_{i = 1}^{i = n} {y_{ij} P,Y_{j} = \frac{{S_{Nj} }}{{S_{D} }}_{i} } \hfill \\ \end{gathered} \right.,$$

where *X* is the network input variable,* X*_*i*_ is the learning sample corresponding to the *i*-th neuron, and *σ* is the smoothing factor. *S*_*D*_ is the model layer summation, *S*_*Nj*_ is the model layer weighted summation, and *Y*_*jk*_ is the prediction result.

In the calculation process, two types of data need to be obtained and analysed, namely, data for the tunnel vault surrounding rock and the settlement time sequence at a given surface point. At the same time, to avoid the influence of sudden changes in the data caused by the short-term rapid construction of shallow tunnels on future deformation prediction, we introduce EMD to decompose the deformation and settlement data into regular modal components. Furthermore, we transform the measured data into a superposition of modal functions via deep learning with the GRNN^34^. Since the key parameter smoothing factor of the GRNN is difficult to determine and subjectively set, we introduce the SSA for intelligent optimization and analyse and process each component and residual of the GRNN. Finally, we construct a tunnel deformation prediction model using EMD-SSA-GRNN and reconstruct the total predicted value by superimposing each predicted value. The sedimentation value function curve conforms to the following equation:

11 $$F(t) = IMF_{1} (t) + ... + IMF_{k} (t) + Res(t),$$

where *Res*(*t*) is the residual.

To remove the excessive noise signal due to modal superposition during EMD, Gaussian white noise is attenuated by reconstructing all the IMFs except for the first IMFs. We then circularly shift the samples of the first IMF and superimpose them with the remaining IMFs while retaining the target signal. We require this process to be repeated until the noise signal is satisfactorily mitigated.

## Preprocessing

### Data acquisition

**Figure 2:**
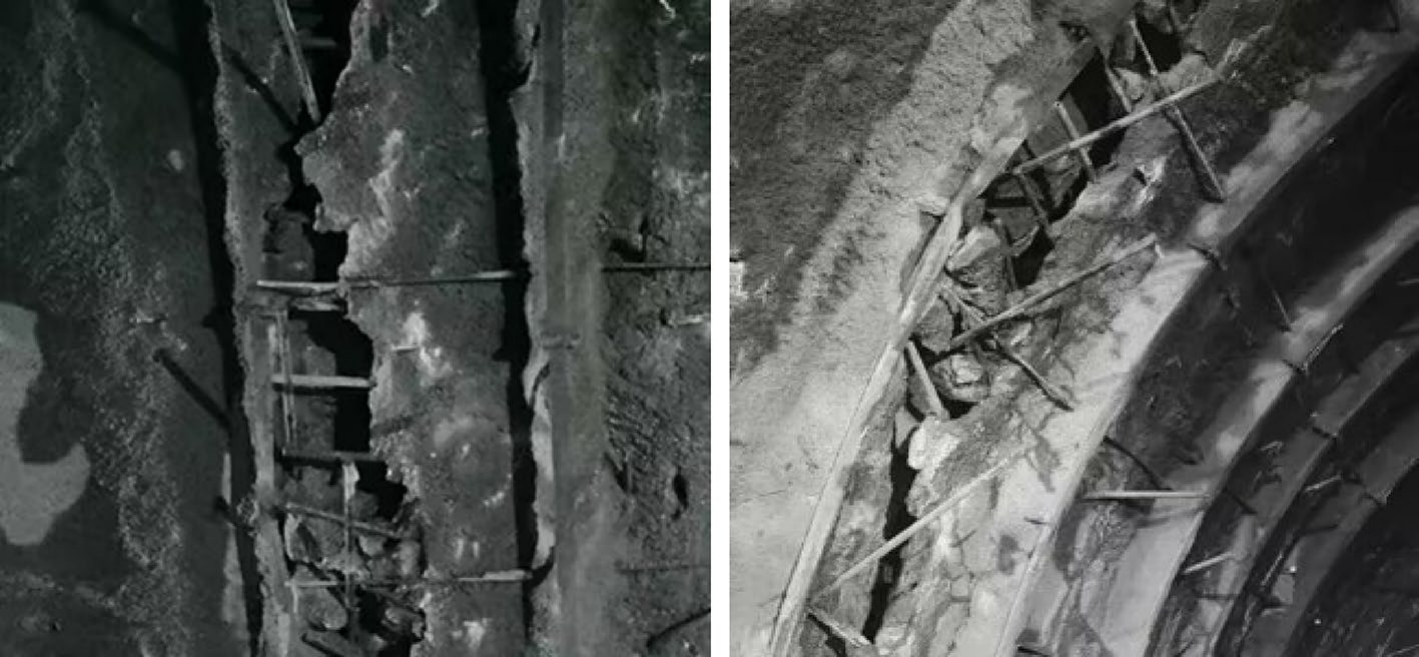
Actual image of the partial collapse of the tunnel.

The measured settlement deformation data were obtained from the tunnel inlet section and cross section of the Kaizhou-Yunyang Highway in Chongqing, China. The tunnel is a separate and independent double-length tunnel, and all the inlets are constructed with bamboo-type cavern doors. The left tunnel inlet pile number is ZK55 + 007.734, and the exit pile number is ZK57 + 424, spanning 2414.266 m. The right tunnel inlet pile number is YK55 + 016.00, and the exit pile number is YK57 + 450, spanning 2434 m. According to site borehole surveys and engineering geological investigations, the tunnel inlet is characterized by tectonic denudation and the erosion of the low mountainous terrain in the area. The stratigraphic lithology is supported by the establishment of a new artificial accumulation layer in the Quaternary system, which includes silty clay with crushed stone, crushed stone soil, argillaceous limestone and limestone intercalated with shale. There are many joints, fissures and seriously weathered rock masses at the entrance of the tunnel. Moreover, a large amount of weak surrounding rock is distributed inside the tunnel. As shown in Fig. 2, the excavation faces of several tunnels are characterized by poor interlayer bonding in the surrounding rock, severe joint development and water seepage, which are serious construction safety hazards.

The focus of existing studies has mainly been on the prediction of long-term deformation after the completion of construction. However, short-term deformation is an important factor affecting construction time and project quality in many tunnels. To clarify the effects of different parameters on different sections of the tunnel during construction, we introduced the EMD-SSA-GRNN algorithm to predict short-term deformation. Moreover, we analysed the prediction accuracy of other models and compared those models with our model.

**Figure 3:**
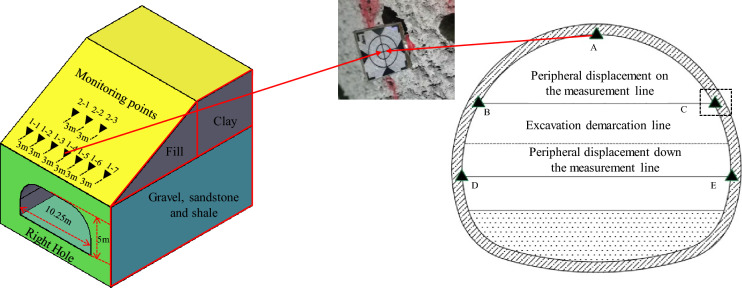
Layout of the surface survey points and the rock surrounding the vault.

To obtain field data, we monitored the changes in displacement at surface points in the tunnel portal section and at various points in the perimeter rock at the tunnel vault. We measured the displacement of a reflective sticker at a centre cross-section point to determine the in situ soil displacement caused by tunnel construction. Additionally, we calculated the changes in the coordinates of neighbouring monitoring points every two days to monitor deformation. As shown in Fig. 3, Measurement Point A is a rock settlement observation point located in the tunnel vault section, Measurement Point BC is an upper-layer convergence observation point, and Measurement Point DE is a lower-layer convergence observation point.

**Figure 4:**
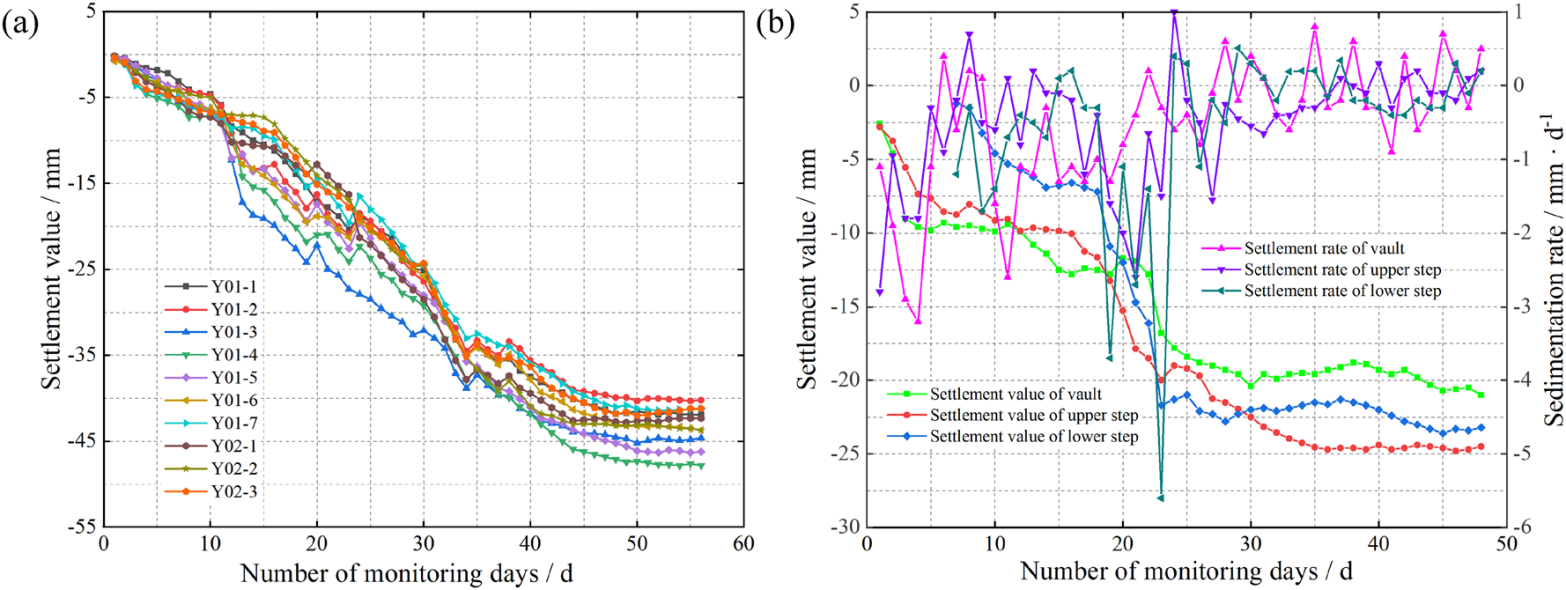
Cumulative sedimentation change curve. (**a**) At the surface of the inlet section of the right cave (**b**) At the rock surrounding the vault of the YK68 + 376 section.

We chose the deformation measured at two locations in the tunnel, namely, at the surface point of the right hole in the tunnel inlet section and at the perimeter rock of the arch in the YK68 + 376 section, as the focus of the study. The number of monitoring days at the surface point of the right tunnel was 56 days, and the number of monitoring days for the YK68 + 376 section was 48 days. The monitoring frequency was 1 time/d. The results obtained from the actual monitoring of both parameters are shown in Fig. 4.

The inlet section of the right cavern of the tunnel is classified as V-class surrounding rock, with a poor bearing capacity and low deformation resistance. Additionally, the layers above and below the tunnel opening are subjected to large disturbances during mechanical excavation. As shown in Fig. 4a, the maximum daily settlement at Monitoring Point Y01-4 along the centreline of the right tunnel inlet reached − 5.6 mm from Day 1 to Day 10 of the monitoring. This was the maximum value among those observed at surface monitoring points in the right cave, and the rate of settlement increased linearly with time. Figure 4b shows that the settlement deformation of the surrounding rock at the tunnel vault can be divided into three main stages: the first 10 days (initial stage), 20 to 30 days (middle stage), and 30 days and after (late stage). In the initial stage, the unstable support of the surrounding rock after tunnel excavation results in a large deformation rate. After completing the primary lining of the upper layer, the settlement deformation of the surrounding rock at the tunnel vault gradually stabilizes. In the middle stage, construction operations such as excavation of the lower layer, establishment of sidewalls and establishment of superelevation arches are carried out, leading to a further increase in the rate of settlement deformation. In the late stage, the structure of the tunnel section gradually stabilizes, and the settlement deformation trend enters a phase of smooth change.

### EMD decomposition

**Figure 5:**
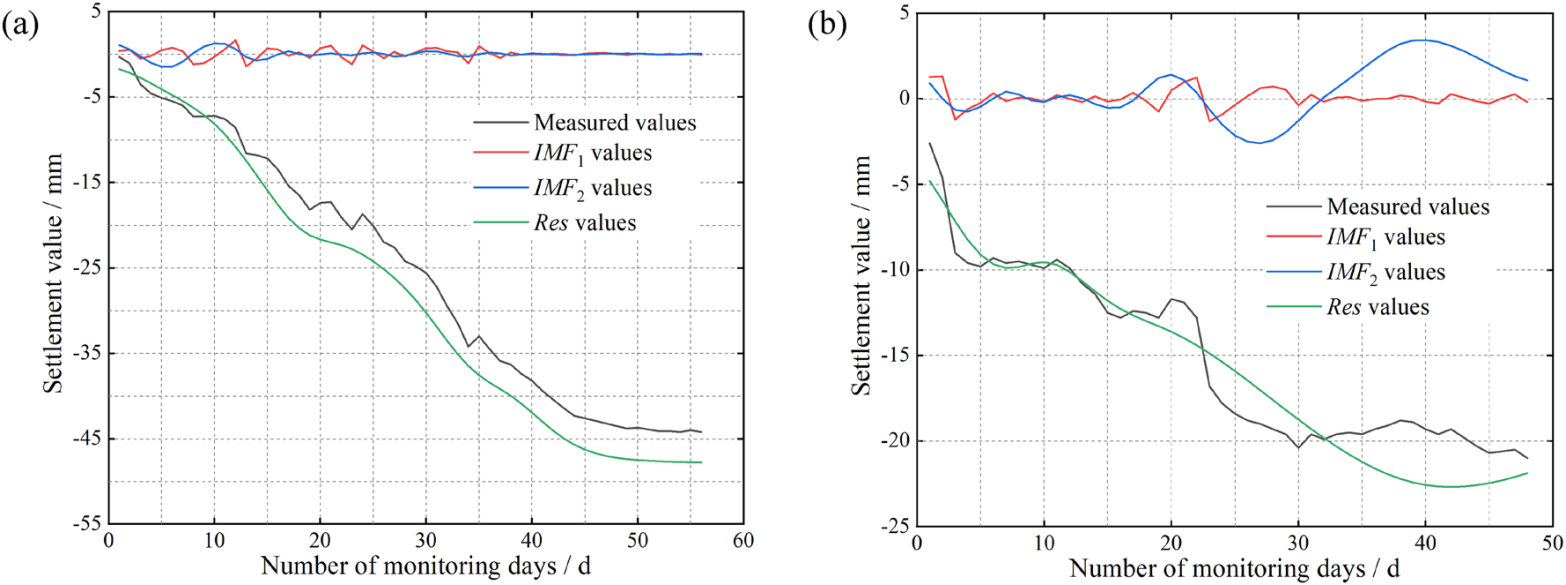
Plots of the intrinsic mode components and residuals. (**a**) At the surface of the inlet section of the right cave (**b**) At the rock surrounding the vault of the YK68 + 376 section.

After monitoring the measured data, we perform decompose the vault monitoring data obtained at surface points Y01-4 and YK68 + 376. As shown in Fig. 5, the decomposed tunnel settlement and deformation data are divided into three parts: the high-frequency component *IMF*_1_, medium-frequency component *IMF*_2_ and low-frequency component residual *Res*. *IMF*_1_ is a high-frequency component that reflects the noise in the tunnel settlement deformation data. Fluctuations in *IMF*_1_ are caused mainly by factors such as the external environment and errors generated by the measurement instruments. *IMF*_2_ is a medium-frequency component that displays a large degree of fluctuation. Fluctuations in *IMF*_2_ are subject to a variety of factors, such as construction techniques, support methods, geologic conditions, and temporal and spatial effects. *Res* is a low-frequency component with relatively smooth fluctuations and can thus reflect the essential characteristics of tunnel settlement deformation. Notably, the data that fluctuate up and down in Fig. 5 are not the settling values but the IMF components, and the settling curves are the descending curves in black and green.

As shown in Fig. 5a, we can obtain two intrinsic mode components, *IMF*_1_ and *IMF*_2_. The curves of *IMF*_1_ and *IMF*_2_ basically satisfy the requirements of the intrinsic mode components regarding the overzero point and the local extremum point. The value domain is basically symmetrical about the X-axis and is depicted as an up-and-down oscillating curve. The residual characteristic curve reflects the approximate characteristics of the original time series data. From the frequency of the IMF curves, the vibration frequency of *IMF*_1_ to *IMF*_2_ continues to decrease, and the curve shows a flat trend. That is, the EMD basically result generally reflects the frequency decrease of the original time series, as supported by deep learning with the neural network. As shown in Fig. 5b, the decreasing trend of the two intrinsic mode components here is consistent with that in Fig. 5a. This shows that the EMD strategy is consistent for various types of data, including settlement feature data. The decomposed *IMF*_1_ and *IMF*_2_ meet the conditions of IMFs decomposition, and their corresponding value domains are basically symmetric about the X-axis, represented as upward- and downward-vibration curves.

Moreover, the fluctuations in *IMF*_1_ and *IMF*_2_ are approximate sinusoidal functions, which reflect the results of neural network learning and the development of settlement. The above analysis shows that EMD can be used to decompose settlement data effectively into intrinsic mode components and decompose intrinsic mode components from high to low with relatively regular variation. Therefore, we find that there is no need to set the number of decomposition modes in EMD. Settling datasets with similar patterns are processed with similar decomposition strategies and similar numbers of modal components. On the other hand, decomposed modal components are influenced by the original signal, resulting in more sudden changes in subsidence and greater volatility. This indicates that the tunnel deformation time series has multiscale characteristics.

### Relevance analysis

According to the on-site construction situation, we selected ten influential parameters, namely, span *B*, height *H*, burial depth *D*, compression modulus *E*, cohesion *c*, internal friction angle *φ*, distance from the palm surface* S*, critical time *T*, and the deformation values *P*_1_ and *P*_2_ at neighbouring measurement points. The data for some of the measurement points are shown in Tables 1 and 2.

**Table 1:** Settlement deformation-related parameters (partial) measured at surface point Y01-4.

Measurement point	*B* (m)	*H* (m)	*D* (m)	*E* (MPa)	*c* (kPa)	*φ* (°)	*S* (m)	*T* (d)	*P*_1_ (mm)	*P*_2_ (mm)	*X* (mm)
Y01-4	10.25	5	1.8	1.7	15	20	2.4	1	− 0.4	0	− 0.3
	10.25	5	1.8	1.7	15	20	4.8	2	− 0.8	0	− 1.1
	10.25	5	1.8	1.7	15	20	7.2	3	− 1.3	0	− 3.5
	…	…	…	…	…	…	…	…	…	…	…
	10.25	5	1.8	1.7	15	20	96.6	46	− 44.9	− 46.1	− 47.8
	10.25	5	1.8	1.7	15	20	98.4	47	− 44.8	− 46.3	− 47.6
	10.25	5	1.8	1.7	15	20	100.2	48	− 44.6	− 46.2	− 47.8

**Table 2:** Vault settlement deformation-related parameters (partial) measured in the YK56 + 376 section.

Measurement point	*B* (m)	*H* (m)	*D* (m)	*E* (MPa)	*c* (kPa)	*φ*(°)	*S* (m)	*T* (d)	*P*_1_ (mm)	*P*_2_ (mm)	*X* (mm)
YK56 + 376	10.25	5	12.03	5.48	28	15	2.4	1	− 2.8	0	− 2.6
	10.25	5	12.03	5.48	28	15	4.8	2	− 3.75	0	− 4.6
	10.25	5	12.03	5.48	28	15	7.2	3	− 5.55	0	− 9.0
	…	…	…	…	…	…	…	…	…	…	…
	10.25	5	12.03	5.48	28	15	110.4	46	− 24.8	− 23.24	− 20.6
	10.25	5	12.03	5.48	28	15	112.8	47	− 24.7	− 23.44	− 20.5
	10.25	5	12.03	5.48	28	15	115.2	48	− 24.5	− 23.54	− 21.0

To further analyse the correlation between each key factor and the maximum deformation of the supporting piles, we performed grey correlation analysis. The calculation is shown in Eq. (12)^34^.

12 $$r_{i} = \frac{1}{N}\sum\limits_{k = 1}^{N} {\left[ {\frac{\Delta (\min ) + \rho \Delta (\max )}{{\Delta_{i} (k) + \rho \Delta (\max )}}} \right]} ,$$

where *r*_*i*_ is the correlation of key factors, *N* is the number of factors, *Δ* (min) is the minimum difference at the second level,* Δ* (max) is the maximum difference at the second level, and *ρ* is the discrimination coefficient.

**Figure 6:**
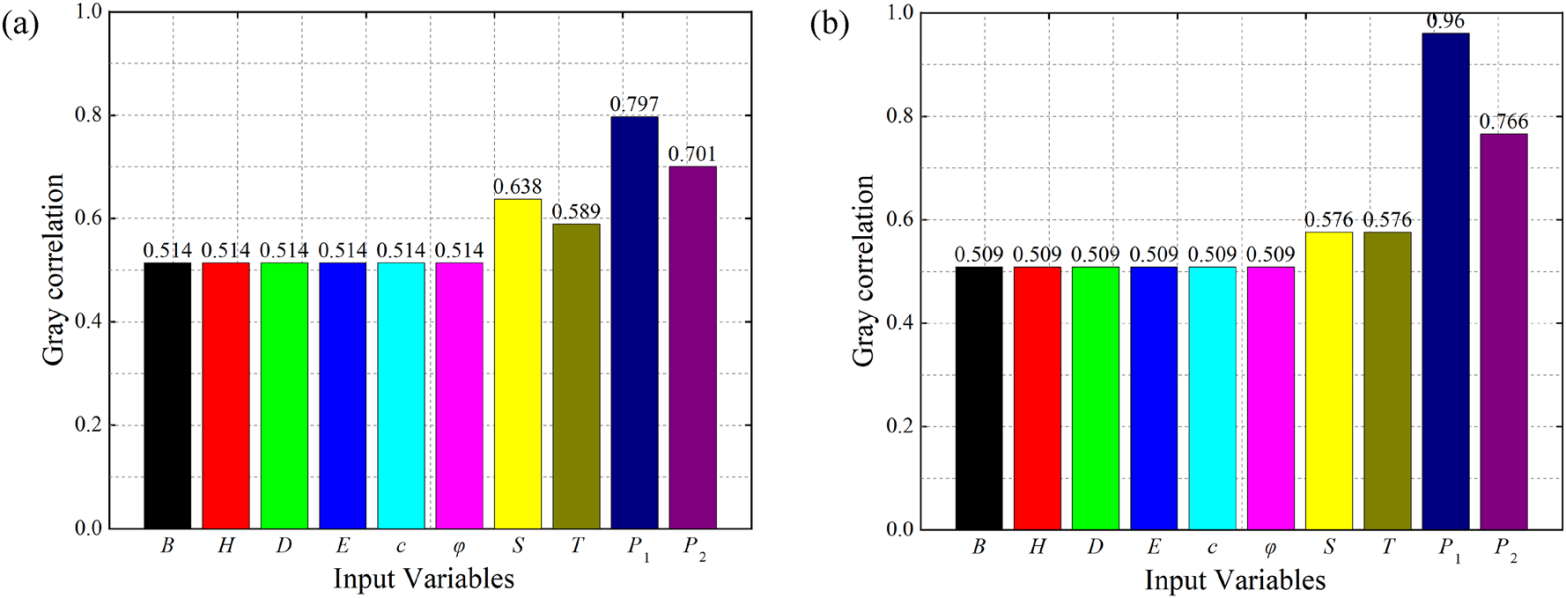
Grey correlations between different parameters and settlement deformation. (**a**) At the surface of the inlet section of the right cave (**b**) At the rock surrounding the vault of the YK68 + 376 section.

To ensure a strong correlation between the input and output variables and a simple model structure, we need to quantitatively validate the results. We evaluated the correlation between each input variable and the settlement at the surface and vault points based on gray correlation. The correlations of the different parameters are shown in Fig. 6.

As shown in Fig. 6, the gray correlation values of the above ten parameters are all greater than 0.5. This means that the correlations between these parameters and the deformation at the surface and vault points are high. At the same time, the correlation values for the distance from the palm surface *S* and the critical time *T* and the deformation values at neighbouring measurement points *P*_1_ and *P*_2_ are significantly greater than those for other related factors. These findings verify that the deformation of the ground surface and the surrounding rock has obvious spatial and temporal effects during the tunnel construction process. Therefore, it is of practical significance to establish a dataset of tunnel deformation using the ten influencing factors selected in this paper.

## Analysis and discussion

Tunnel construction is characterized by a high speed and complex construction conditions underground. To accurately predict future vault deformation in a timely manner, we use a single-step prediction method to construct a prediction model with a 5-dimensional input and a single-dimensional output. Considering that the total data volume is relatively low, we set the ratio of the data volume between the training set and the prediction set to 8:2. This means that at surface point Y01-4, data from the first 44 days of monitoring constitute the training set, and data from the last 12 days of monitoring constitute the prediction set. At the top of the arch in section YK56 + 376, data from the first 38 days of monitoring constitute the training set, and data from the last 10 days of monitoring constitute the prediction set. With the training set and prediction set data, we can compare the prediction accuracies of the GRNN, SSA-GRNN, EMD-GRNN and EMD-SSA-GRNN models. In the GRNN and EMD-GRNN models without SSA, the smoothing factor is set to the default value of 1 or 0.1. In the SSA-GRNN and EMD-SSA-GRNN models with SSA, the SSA is used for the intelligent selection of smoothing factors. Notably, in SSA, the sparrow population is 20, the maximum number of iterations is 50, the optimization parameter is the smoothing factor, the variable dimension is 1, the safety threshold is 0.8, the proportion of finders is 0.7, and the proportion of sparrows aware of danger is 20%. The parameters of the base GRNN model are selected as shown in Table 3.

**Table 3:** Base parameters of the GRNN model.

Neural network model	Number of neurons	Number of layers	Number of iterations	Number of epochs
GRNN	10	4	20	64

**Figure 7:**
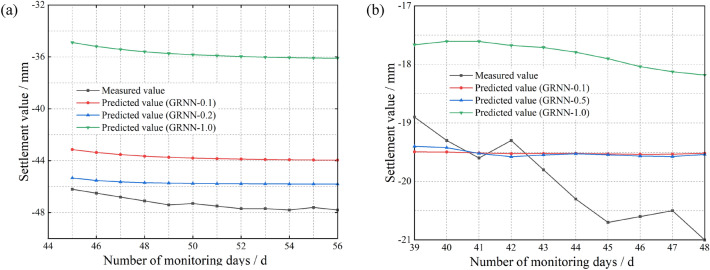
Prediction results of the single GRNN model. (**a**) At the surface of the inlet section of the right cave (**b**) At the rock surrounding the vault of the YK68 + 376 section.

First, we utilize a single GRNN model with different smoothing factors for single-step prediction. The prediction results of the single GRNN model are shown in Fig. 7.

As shown in Fig. 7, we find that the results of the single GRNN model differ significantly from the actual measured data, indicating low prediction accuracy. Notably, the GRNN-1.0 predictions differ more from the measured values than do the GRNN model predictions with various smoothing factors. Therefore, when using the GRNN model to carry out tunnel settlement prediction analysis, the value of the smoothing factor cannot be directly set to 1.0. Moreover, it is difficult to capture the data during some mutations because the time series information is limited. This leads to smooth and less smooth predictions at various iterations.

**Figure 8:**
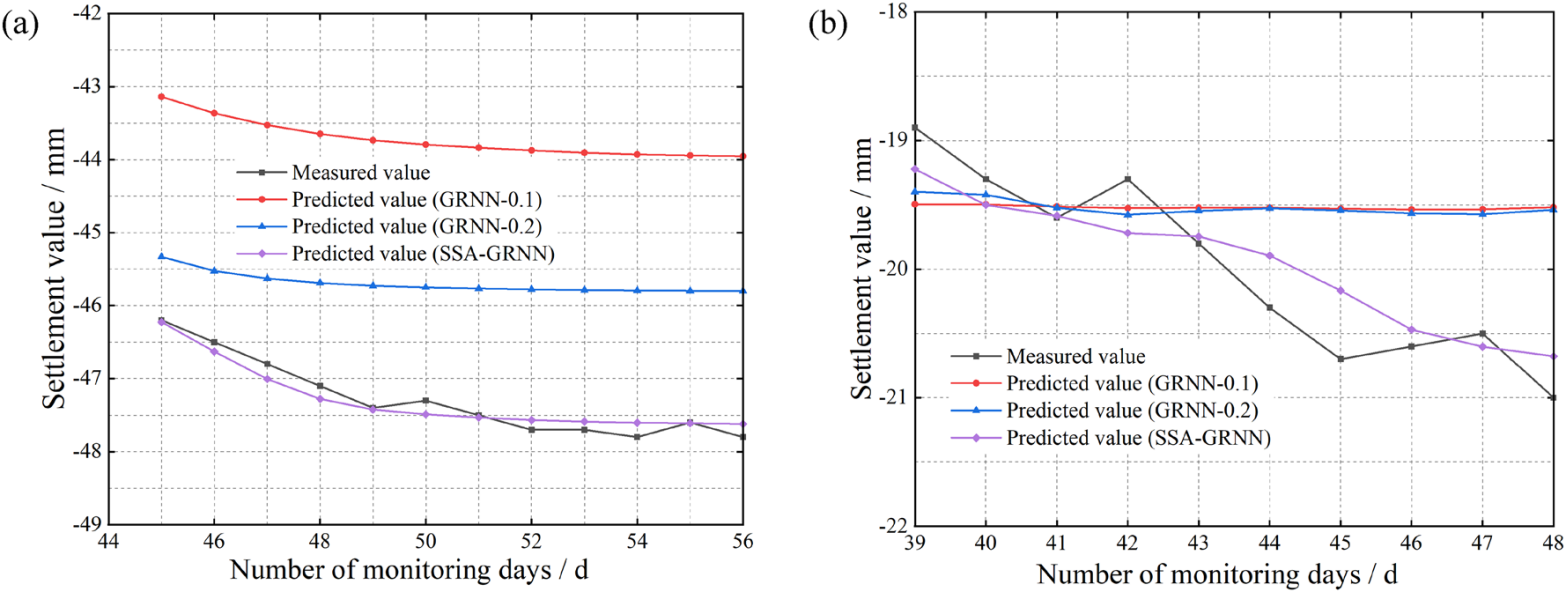
Comparison of the prediction results of the single GRNN model and the SSA-GRNN model. (**a**) At the surface of the inlet section of the right cave (**b**) At the rock surrounding the vault of the YK68 + 376 section.

Second, we introduce the SSA to determine the optimal smoothing factor and analyse the prediction results of the single GRNN model and SSA-GRNN model for comparison. The predictions of the two models are shown in Fig. 8.

As shown in Fig. 8, the overall prediction accuracy of the SSA-GRNN model is higher than that of the single GRNN model. This is because a single GRNN model tends to output results quickly during training if the smoothing factor is directly set to 0.1 or 0.2. Consequently, the prediction accuracy is unacceptable for complex deformation settlement data. Therefore, the absence of SSA optimization for the smoothing factor leads to a significant decrease in the prediction accuracy of the GRNN model. Based on the prediction accuracy of these two models, we believe that the SSA plays an important role in enhancing the prediction accuracy.

**Figure 9:**
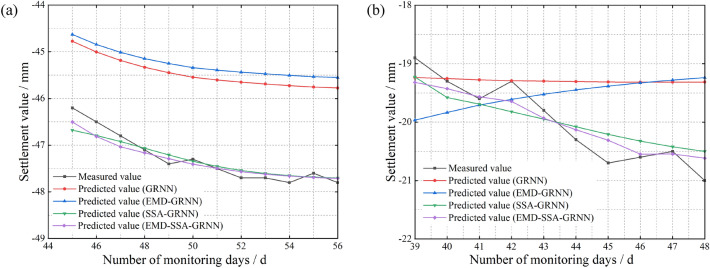
Comparison of the prediction results of the SSA-GRNN model and the EMD-SSA-GRNN model based on gray correlations. (**a**) At the surface of the inlet section of the right cave (**b**) At the rock surrounding the vault of the YK68 + 376 section.

Finally, we decompose the input data via EMD and subsequently construct the EMD-SSA-GRNN prediction model based on an analysis of multiple influencing factors. The prediction results of the four considered models are shown in Fig. 9.

To verify the prediction accuracy of the different models, we use the following three evaluation metrics: mean square error (MSE), mean absolute error (MAE), and root mean square error (RMSE). The expressions of these three comprehensive evaluation indicators are as follows:

13 $${\text{MSE}} = \frac{{\sum\nolimits_{i = 1}^{n} {(r_{i} - p_{i} )^{2} } }}{n}$$

14 $${\text{MAE}} = \frac{{\sum\nolimits_{i = 1}^{n} {\left| {r_{i} - p_{i} } \right|} }}{n}$$

15 $${\text{RMSE}} = \sqrt {\frac{{\sum\nolimits_{i = 1}^{n} {\left( {r_{i} - p_{i} } \right)^{2} } }}{n}} ,$$

where *p*_*i*_ is the predicted value, *r*_*i*_ is the actual value, *n* is the number of data points, and *i* is the number of iterations.

The error evaluation metrics for the four models in Fig. 9 are shown in Table 4.

**Table 4:** Prediction model error comparison.

Measurement points	Predictive model	Training time (s)	MSE (mm^2^)		RMSE (mm)		MAE (mm^2^)		*R*^2^
			Time series	Influencing factors	Time series	Influencing factors	Time series	Influencing factors	
Y01-4	GRNN	11.2	0.6407	0.9275	0.8004	0.9631	0.6578	0.7750	0.755
	EMD-GRNN	13.5	0.3885	1.0272	0.6233	1.0135	0.5411	0.8716	0.804
	SSA-GRNN	16.8	0.1343	0.1343	0.3664	0.3665	0.3189	0.3189	0.851
	EMD-SSA-GRNN	18.1	0.0471	0.0762	0.2171	0.2759	0.1797	0.2299	0.975
YK56 + 376	GRNN	9.5	2.6596	3.3801	1.6308	1.8385	1.5858	1.8265	0.742
	EMD-GRNN	11.7	6.4696	4.1522	2.5435	2.0377	2.5079	2.0243	0.792
	SSA-GRNN	15.2	0.0191	0.0369	0.1383	0.1922	0.1178	0.1494	0.855
	EMD-SSA-GRNN	17.4	0.0548	0.0276	0.2342	0.1662	0.2036	0.1388	0.984

According to Fig. 9 and Table 4, the EMD-SSA-GRNN model based on multiple influential factor analysis yields the highest prediction accuracy compared to the other three models. Focusing on the prediction curves of the EMD-GRNN and GRNN models, we find that both exhibit linear trends in the later stage of prediction. Focusing on the prediction curves of the EMD-SSA-GRNN and SSA-GRNN models, we find that the results of the EMD-SSA-GRNN model are more accurate than those of the SSA-GRNN model. By comparing the above results, we can clarify the contributions of the SSA and EMD to improving the prediction accuracy and can draw clear conclusions about the EMD-SSA-GRNN model constructed in this paper. That is, the EMD-SSA-GRNN model has three advantages: the ability to characterize the trend in fluctuation periods, provide accurate predictions in the late prediction stage, and produce results close to the actual values.

**Figure 10:**
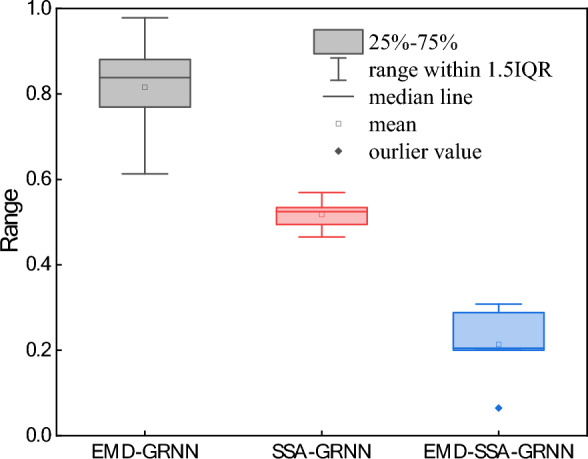
Box plots for the different models.

Additionally, to show the dispersion of the data used during training for the different models, we constructed box plots for the predicted models. The box plot information is shown in Fig. 10.

## Conclusions

1. The initial settlement rate of tunnel deformation is significantly lower than the mid-term settlement rate but much greater than the final settlement rate. The surface settlement at the midline of the tunnel entrance section is the largest, and the further away from the tunnel centre a point is, the smaller the surface settlement deformation is. The settlement deformation and convergence trends of the vault of the internal section of the tunnel are consistent with those at the entrance section, both of which are obviously affected by the excavation of the upper and lower geological layers.
2. The prediction results of different models are compared and verified with tunnel-measured data, and it is found that by using the SSA to optimize the smoothing factor for the key parameters of the GRNN model, compared with the GRNN model, the SSA-GRNN model can significantly improve prediction performance. Compared with that of the GRNN model, the prediction accuracy of the SSA-GRNN model is improved by 72% and 94% in terms of time series prediction and multifactor prediction, respectively.
3. The correlation degree analysis method is used to analyse the factors affecting surface subsidence and surrounding rock deformation, and datasets of 10 factors affecting tunnel deformation, such as the distance from the palm face, air-facing time, and burial depth, are established. Compared with that of other models, the prediction accuracy of the EMD-SSA-GRNN model based on multiple influencing factors improved by 19.2% to 59.4%. The combined EMD-SSA-GRNN prediction model can avoid the interference of short-term mutation data on the prediction of the overall settlement trend, and the agreement between predictions and monitoring data is significantly better than that for other combined prediction models, reflecting better applicability and stability.
4. The combination of soft carbonaceous slate and super high ground stress has made large deformation in soft rock a common worldwide problem in the history of tunnel construction. Compared with those of other deformation prediction models, the prediction results of the EMD-SSA-GRNN model are more advantageous in terms of accuracy, and this model is faster to run than other models. Therefore, the application of this algorithm for short-term large deformation prediction during tunnelling will be the focus of our future research. The EMD-SSA-GRNN model constructed in this paper still needs to be further improved in terms of parameter optimization. Moreover, in future research, we can consider implementing the SSA in the grid search algorithm to establish a method capable of automatic search and optimization.

## Data Availability

If someone wants to request the data from this study, please contact the author Jiajun Shu.
